# Why and how to set up a Bioinformatics Learning Lab (BILL)

**DOI:** 10.1371/journal.pcbi.1013236

**Published:** 2025-08-28

**Authors:** Anna-Sophie Fiston-Lavier, Emira Cherif, Marie-ka Tilak, Arnaud Soulier, Catherine Breton, Anthony Boureux, Remy Costa, Xavier Miahle, Clothilde Chenal, Floris Schatt1, Camelia Sennaoui, Jean-Christophe Avarre, Anne-Sophie Gosselin-Grenet

**Affiliations:** 1 ISEM, Univ Montpellier, CNRS, IRD, Montpellier, France; 2 Institut Universitaire de France (IUF), Paris, France; 3 Computer science dept, Univ Montpellier, France; 4 Alliance Bioiversity CIAT Europe - Montpellier Office, Bioversity International France, Montpellier, France; 5 French Institute of Bioinformatics (IFB)-South Green Bioinformatics Platform, Bioversity, CIRAD, INRAE, IRD, Montpellier, France; 6 IRMB U1183, Univ Montpellier, Inserm, Montpellier, France; 7 IGH, Univ Montpellier, CNRS, Montpellier, France; 8 SeqOne, Montpellier, France; 9 MIVEGEC, Univ Montpellier, CNRS, IRD, Montpellier, France; 10 DGIMI, Univ Montpellier, INRAE, Montpellier, France; SIB Swiss Institute of Bioinformatics, SWITZERLAND

## Introduction

In the field of education, innovative teaching methodologies have indisputably gained prominence in recent years to enhance student engagement and comprehension. “Active Learning” is a broader concept that encompasses various techniques designed to involve students in the learning process actively. They are actively engaged rather than passively receiving information, taking part in discussions, group activities, and hands-on exercises during class. Active learning is an effective method for developing critical thinking, problem-solving skills and collaboration. It emphasizes the application of knowledge in real-world scenarios such as a research laboratory. By fostering collaboration and hands-on experiences, active learning is supposed to promote a deeper understanding of concepts and a better retention of information. It encourages students to take ownership of their learning process [1]. In most research laboratories and companies, scientists need to collaborate with experts from other fields to complement and expand their own skills. Nowadays, it is almost unavoidable to integrate multidisciplinary expertises. In bioinformatics education, it is crucial to prepare students for such multidisciplinary collaboration with professionals across diverse domains such as biologists, ecologists, physicians, statisticians, or physicists. By fostering these connections, such bioinformatic classes could also serve as a bridge between the academic and research worlds, equipping students with the skills needed for effective teamwork and collaborative problem-solving. Another challenge in bioinformatics education is to keep the teaching classes up to date while this field is evolving rapidly specifically in sequencing technologies and in terms of algorithms to analyse the sequencing data [2, 3]. For example, advances in sequencing technologies are driving down the sequencing costs, leading to an impressive increase of genomics data and projects. It has now become increasingly accessible, generating large volumes of sequencing data. However, processing and analysing this data remain challenging due to the substantial computational resources and specialised expertise required. There is, therefore, an urgent need to train new generations of students to be computationally proficient in the latest algorithms and approaches for handling and analysing these sequencing data, ensuring keeping pace with technological developments in the field [4]. Salazar *et al*. proposed in 2020 a guide for the training of teachers, biologists and students specifically dedicated to analysing Oxford Nanopore long-read sequencing data [4]. It is essential to regularly update knowledge and skills to maintain students’ employability. A number of pedagogical initiatives have recently been developed with the objective of promoting collaborative research projects and linking education and research in the field of bioinformatics. One well-known educational competition is the International Genetically Engineered Machine (iGEM; https://igem.org/) competition, which is an international competition on synthetic biology. In each city worldwide, a group of students engages in a collective research project, from the design to the presentation of the experiments and results. Each interdisciplinary group of students is afforded the opportunity to use and combine their respective skills in order to engage in collective research project development. Subsequently, the participants are invited to present their research project at the annual Jamboree. More recently, local educational initiatives have emerged, including Meet-U [5], a student competition established in 2017. Each group of students from the bioinformatics master’s program in Paris, France, investigates an unsolved biological question, and then presents their analyses and results to a panel of experts comprising teachers and researchers. Meet-U places particular emphasis on structural bioinformatics, with a specific focus on protein-protein docking [5]. Consecutive to the success of this initiative, a European competition was launched in 2021 called Meet-EU (https://www.hdsu.org/meet-eu-2021/). In order to ensure the long-term viability of such initiatives, it is essential to establish dedicated timeframes, locations and resources to facilitate interactions and collaborations among bioinformatics students, enabling them to leverage their diverse skill sets and become active agents in their own educational development. In line with these initiatives, we proposed to develop an innovative interdisciplinary learning laboratory: The acronym BILL stands for BioInformatics Learning Lab. BILL is an innovative teaching project dedicated to bioinformatics based on active learning and simulation of a research laboratory for students from the University of Montpellier in France. Gathering students from microbiology and bioinformatics fields, BILL project offers a collaborative space consisting of two laboratories dedicated to sample preparation, including DNA extraction, library preparation and sequencing (“wet lab”), which open onto two laboratories dedicated to secondary and tertiary bioinformatics analyses (“dry lab”). These rooms give students the opportunity to engage with a real laboratory, enabling them to generate and analyse data themselves in order to address research questions collectively. This project has to be supported by a dynamic multidisciplinary team of lecturers, researchers, engineers and technicians, from production and analysis of data to the valorization and dissemination of results. In the present article, after explaining the motivations behind the BILL project, we present the elements needed to set up such an innovative teaching project: the location, the teaching approach, the research project, and the short- and long-term organization and valorization.

## Exchange of skills and knowledge between students working together on a joint scientific and teaching project

**Fig 1:**
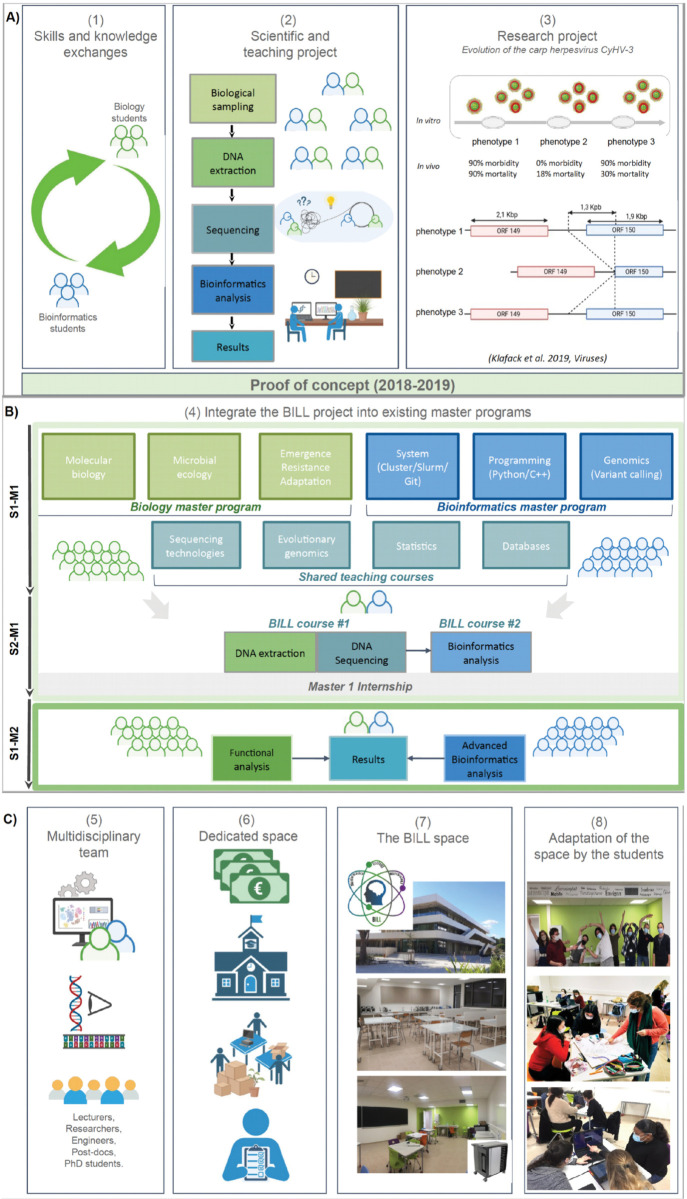
BILL project. A) The project is built on collaboration between bioinformatics and biology students. Its key components include biological sampling, DNA extraction, sequencing, and bioinformatics analysis, supported by the combined efforts of students, researchers, and technical teams. Initial results on CyHV-3 in vitro evolution highlight phenotypes associated with morbidity and mortality variations. A comparative genomic analysis of viral populations from three phenotypes (cell passages) revealed a large deletion of 1,363 bp in the predicted ORF150, associated with loss of virulence (phenotype 2). The first year served as a proof of concept and inspired further work [6, 7]. B) To pursue the project, specific courses were selected and adapted alongside mandatory ones both in the bioinformatics and biology in the master’s programs. During the first semester of the M1 (S1-M1), the students are trained in general concepts in bioinformatics (programming... ) and biology (virology, evolution... ). Then during the second semester of the M1 (S2-M1), the students work in mixed groups on the BILL research project. After the project, they have to do their Master 1 internship. During the second year of the master program (M2-S1), students develop their analytical skills (functional and advanced bioinformatics) to interpret results and prepare publications. C) The BILL project was sustained through shared teaching courses, institutional funding, computational resources, and experimental contributions.

The main objective of the BILL project is to equip bioinformatics and biology students with critical skills in scientific research: effective communication across disciplines. This includes translating biological problems into computational questions and clearly interpreting and presenting bioinformatics results. The initial challenge, therefore, is to bridge the gap between bioinformatics and biology. In 2018, a collaboration has been initiated between the master programs in microbiology and bioinformatics from the University of Montpellier (each involving around 25 students). The objective was to propose a joint project for the nearly 50 students from the two master programs, entailing the combination of skills from biological sampling to bioinformatics analyses (See Fig 1A). During the first edition of this project, students enrolled in the bioinformatics master’s program assumed responsibility for organizing the Montpellier Omics Days (MODs) (see box1), and extending an invitation to their colleagues in the microbiology program to participate in the MODs’ workshop. They trained them how to use Linux command lines for the analysis of short-read sequencing data. Subsequently, using their new bioinformatics skills, the microbiology students were able to start the analyses of the sequencing data obtained in the frame of a research program.

**BOX1 Montpellier Omics Days:** In the frame of the Montpellier Bioinformatics master’s program, the teaching team has, since 2013, encouraged master students to organize a scientific event on omics sciences as part of their curriculum. The field of omics encompasses a wide range of research areas. While some editions have focused on omics in medical research, others have addressed the fields of environmental sciences and evolutionary biology. This event has been called the Montpellier Omics Days (MODs). The idea behind this approach is manifold. From the students’ perspective, the experience offers an opportunity to learn about the behind-the-scenes aspects of organizing a scientific event, including group work, coordination and logistics. Additionally, by choosing the format and program of the event, students can learn about areas of hot topic research in the omics sciences, on top of the basics learned in class. The format of the event is a one-day conference with guest speakers, followed by another day of student-led training courses in omics data analysis. For the past years, the master’s students in bioinformatics have proposed introductory training courses: Linux command lines, R programming, and machine learning for the analysis of sequencing data. As a consequence of technological advances and the expansion of communication with others students, the event began to welcome more than 200 attendees since 2017. The MODs has consequently become a constituent element of the Montpellier science event landscape.

## Comparative analysis of virus genomes: An appealing and evolving research project

In the first collaborative study conducted in 2018, we investigated the in vitro evolution of an emerging virus called Cyvirus cyprinidallo3 (CyHV-3), which causes fatal disease in common carp and ornamental koi carp [6, 8]. Since its emergence in the late 1990s, this 295-kpb double-stranded DNA virus has spread to numerous countries worldwide and caused severe environmental and financial losses in aquaculture industries. We started a comparative genomic analysis of viral populations giving rise to different virulent, avirulent, and intermediate phenotypes (See box2 and Fig 1A). The analysis revealed the presence of a large deletion of 1,363 bp in the predicted open reading frame (ORF) 150, which was detected in the majority of the viruses from the avirulent form, but not in the other viruses. This initial result suggested that CyHV-3 evolves, at least in vitro, through a combination of haplotypes that alternatively become dominant or under-represented, potentially influencing the virulence of CyHV-3. These results were presented at a congress by the students (MicrobiOccitanie 2019; https://microbioc2019.sciencesconf.org), and were then published in a peer-reviewed journal with the students as co-authors in a consortium [6, 9]. Since then, we rethink the project every year in order to include new biological material, new experiments, and consequently investigate new research questions. The proposed research project has to offer a number of significant benefits for the field of education. Firstly, the project must address a large panel of topics such as ecological, health and economic questions, which helps to motivate most of the students. Secondly, the project should be achieved in a matter of weeks, with a clearly defined protocol involving a few steps and a small genome size to sequence. This makes it ideal for incorporation in a teaching course. Thirdly, we can keep up with the evolution of this research project and easily incorporate additional samples and enlarge the scope of the scientific project. Fourthly, the research project has to undergo significant advancements, encompassing both refined research questions and technological enhancements. Finally, the required skills should be multidisciplinary, as the project demands expertise in molecular biology for DNA extraction, bioinformatics for sequencing data analysis and variant calling, statistics for data analysis, and microbiology and cellular biology for tertiary analyses and result interpretation. In such a way, the research project also enables students to apply and showcase their skills to classmates, while also gaining proficiency in new domains.

**BOX2 Research project:** In order to understand the evolution of virulence of CyHV-3, It was proposed to conduct experimental evolution experiments (See Fig 1A). To this end, an isolate of the virus from Taiwan (KHV-T) was serially passaged onto common carp brain (CCB) cells over 100 times at 24^°^C, and the virulence of the virus from selected passages was tested in common carp. For these passages, the DNA of the virus was extracted from the corresponding passages and then sequenced using sequencing technologies. Since a reference genome of CyHV-3 genome is available and the reads generated from the sequencing were mapped against this latter. Finally, the single nucleotide polymorphisms (SNPs) and structural variants (SVs) were computationally extracted and analysed (See Fig 1A and S1 Fig) [6, 9].

## Make the project sustainable by integrating it into the existing master’s program

Following the successful completion of the proof of concept, we established a team of experts, allocated dedicated space and scheduled slots within the master programs and made the necessary adjustments to ensure optimal conditions for the project’s continuation over the years. We first integrated the project in the two master programs. We adapted courses of the first semester of the first year of each master program to make sure that the students would acquire the expected skills before starting the BILL project. To do so, we beforehand identified the mandatory skills required for this new teaching. In the particular case of bioinformatics master students, we used to introduce basics of genomics including how genomic data are generated and how to analyse them (e.g., annotation, variant calling), the variety of genomics file formats. In these algorithms and programming courses, following the theoretical classes, students are invited to participate in projects requiring them to implement a script for parsing a flat file such as mapping (SAM) or variant calling (VCF) file (See Fig 1B). New courses were also proposed, such as courses in sequencing technologies and evolutionary genomics (See Fig 1B). In the second semester of the first year, two BILL courses were introduced: one focused on the DNA extraction and sequencing, and the second on the bioinformatics analysis of sequencing data for the detection and analysis of variants. In these courses, students work in teams on a real research project defined by the teaching team. During the first semester of the second year of the master program, other BILL courses are devoted to completing the analyses, going into greater details and ending with the exploitation of the results. While we ask the bioinformatics students to dig deeper into the quality and reproducibility of the results and how to improve the BILL pipeline (See S1 Fig) [11], we ask the biologists to conduct a more in-depth functional analysis. Therefore, each student continues to work on the BILL research project over 2 years.

## Make it real

In order to achieve this, it was necessary to bring together a teaching team made up of researchers from the different fields involved, teacher-researchers to make the link between research and teaching expectations, engineers for their technical skills in molecular biology or bioinformatics, and PhD students for their experience as former students (See Fig 1C). For the amount of students involved (around 50), we recommend a team of at least four people with two bioinformaticians and two molecular biologists, with the option of welcoming new members every year, if necessary. All members might need to be trained to be updated about the new sequencing technologies every year. Before the BILL courses, the team members have to prepare the material. Then, in order to supervise the students properly, they will meet with them at least twice a week (for three hours each) for two months, ending with a presentation of the results for each group of students. The next challenge was to identify and design a space where we could bring the students together to carry out the various experimental and computational stages of the project, as well as facilitate small group discussions in a friendly environment. We began exchanging ideas with the students and between ourselves in order to think together about the needs for a dedicated space. We designed a molecular biology practical room for DNA extraction and sequencing library preparation, close to a meeting room where we can organise the bioinformatics analyses and allow students to work in groups. Furthermore, we also discussed how to create a learning atmosphere (deco, wall paint, mobile furnishings... ; See Fig 1C). With the financial support of a specific program from the University of Montpellier dedicated to promote innovative teaching projects, we (i) obtained the necessary space in the new building of the Ecology teaching department, (ii) fully equipped two wet practical training rooms to accommodate 50 students and (iii) purchased mobile chairs and tables to adapt the meeting room for working groups or oral presentations. Next, we purchased laptops that were suited to the mobile environment and powerful enough to handle the live sequencing technology, and experimental materials (See Fig 1C). For DNA sequencing, we chose to buy portable sequencers from Oxford Nanopore. This company provided us for free some materials for the two first BILL editions. Such opportunities allowed us to start the BILL project. Later on, we were invited to join the Beta Education Program of Oxford Nanopore that offers resources such as feedback of other teaching projects on the sequencing technologies and discounts for the experimental reagents and kits.

## Implementing best practice in bioinformatics and teaching tools

Furthermore, we had to think about hot storage for computing, and cold storage for backing up data and results. Since student accounts did not allow for the storage of large sequencing datasets, we applied for funding with other courses at the university to buy a shared cluster (https://ngstc.iutms.umontpellier.fr/). For the sequencing part, we had to purchase the molecular biology equipment to extract, quantify and sequence DNA samples. However, it is important to encourage the sharing of equipment from laboratories associated with the project or other courses. Adherence to FAIR (Findable, Accessible, Interoperable, and Reusable) principles is a cornerstone of the educational approach, ensuring that all data and processes are transparent and replicable [10]. We chose to use tools such as Snakemake [9, 11] GitHub platform [12], and Jupyter Notebooks [13] to share the scripts dedicated for the research project. Specifically, we developed a Snakemake pipeline for variant calling (https://github.com/asfistonlavie/BILL; [9]). This approach includes numerous control steps, generates large output files, and integrates visualization and release features to ensure that students can follow updates in real-time (See S1 Fig). It thus supports pedagogical objectives by providing a well-structured approach to teaching the management and analysis of large and complex datasets. In order to test students’ knowledge during the sessions and adapt the following sessions by revisiting certain concepts if necessary, we set up interactive quizzes. To evaluate the knowledge gained from the project, the groups are asked to summarize their results [14]. We ask them to focus on different sub-questions of the project (e.g. variations between technical replicates, variations between runs under different conditions...). Each group also has to present their results orally. In order to take into account both individual participation and teamwork, students are assessed by their teachers on their scientific work (oral and written) and by each member of their group on their participation. For the latter, we provide a marking grid to ensure fairness.

## Dissemination of the BILL project and student achievements

Several key strategies have been implemented to strengthen interaction between research and education. A strong emphasis has been placed on sharing results with the research and education communities. Each year, students are actively encouraged to contribute to scientific publications with the research team. Writing research papers in collaboration with students not only strengthens their academic skills but also prepares them for future careers in research. In addition, participation in conferences such as the MODs (See box1) or national conferences specialised in bioinformatics (such as JOBIM - French bioinformatics conference) or in virology (JFV - French Virology Days) offers students an opportunity to present the BILL project, their work and to expand their professional networks. On top of that, participating in such events gives both students and fellow researchers access to cutting-edge knowledge and tools. Keeping up with emerging technologies is essential for remaining at the forefront of education, ensuring that each year brings new ideas, updated methodologies, and cutting-edge tools to the curriculum. In addition to the formal academic environment, other educational initiatives, such as the production of educational videos or the participation in public events like “Fête de la Science,” helps to broaden public understanding of genomics and related fields. Furthermore, networking through a website, wiki, blog, and GitHub repository fosters a collaborative environment, allowing the community to grow and stay connected. Finally, it is essential to get a sense of the impact of such teaching innovations on students’ knowledge and skills. Even if it is complex to estimate, we can assess the impact of this pedagogical innovation by the number of students who pursue their career (internship, thesis, job, etc...) in the project field of study. Feedback from internship supervisors can also help to assess students’ independence and comfort with the tools, while feedback from the students themselves provides insight into how helpful BILL has been in facilitating their learning.

## Challenges

When implementing innovative educational approaches, the primary challenge lies in ensuring the project’s sustainability. This involves maintaining an evolving research framework with new, high-quality questions each year. It also implies dealing with the unpredictable nature of research projects. For example, if sequencing experiments fail, having a contingency plan ("plan B") is crucial to ensure the course objectives are still met. This adds a layer of complexity to both course planning and delivery. Financial stability is also critical and requires consistent funding over years. Assuming that the main scientific, sequencing and computing equipments are already available, the €3,000 annual running cost includes kits and laboratory consumables but does not include the salary of the teaching team. To foster hands-on, project-based learning, we limit class sizes to 45–50 students, though smaller groups pose financial challenges as fixed costs remain comparable. A core motivation for these new educational methods is to bridge the gap between education and research. Courses need to be frequently updated to include the latest scientific concepts and theories, sequencing technologies, data analysis methods and molecular biology techniques to keep students employable. This need for constant updating can place an undue burden on teachers. This unconventional approach to education can be destabilizing for students. Many are unaccustomed to collaborative interdisciplinary works with peers from different academic backgrounds, which can make communication challenging. In addition, students often have limited exposure to the world of research and its expectations, such as publishing scientific papers, communicating research results, and understanding the competitive nature of academic achievements. The course is tailored to fit specific project requirements, each year, in terms of sequencing, NGS technologies, or data analysis. Adapting course content to these different needs is therefore essential. Ensuring the students develop the necessary competencies within a short time frame requires flexibility in teaching and careful curriculum design. Despite these hurdles, these innovative methods provide valuable opportunities for students to immerse themselves in the world of research while developing essential problem-solving skills. Additionally, such a training project raises students’ awareness of important and timely topics, such as the carbon footprint of research activities [15].

## Perspectives

By addressing the challenges of teaching students with diverse backgrounds, and the unpredictability of research, teachers can create impactful learning experiences that prepare students for careers in a rapidly evolving field such as genomics. However, longevity of such learning methods requires continuous adaptation and strategic planning, and commitment to excellence. The BILL project is still in its early stages, making it challenging to assess its impact on the employability of students. However, initial feedback from graduated master students has been highly encouraging, suggesting positive outcomes. To better understand and quantify the project’s influence, it will be valuable to establish metrics for evaluating its long-term impact. These metrics could include graduate employment rates, career progression, and skill application in professional settings, providing insight into the effectiveness of this innovative educational approach. As the BILL project progresses, an important next step will be the development of a dedicated database to store data and results from previous years. This will facilitate longitudinal analyses, enable reproducibility, and enhance the project’s scalability. Additionally, the project could evolve to explore new research themes, broadening its scope and fostering interdisciplinary collaborations. Initiating a research project on a novel topic would not only diversify the project’s portfolio but also provide students with opportunities to address emerging scientific challenges, further enriching their educational experience.

## Supporting information

S1 Fig Snakemake Pipeline for variant calling designed in the BILL project. The pipeline has been designed by the supervisors of the project and collaboratively developed with students. The BILL team then continues to maintain and update the pipeline (8).
(TIF)

## Open Access

For the purpose of Open Access, a CC-BY public copyright licence has been applied by the authors to the present document and will be applied to all subsequent versions up to the Author Accepted Manuscript arising from this submission.
