# Supplementary figures and images for: Why and how to set up a Bioinformatics Learning Lab (BILL)
Source: PLoS Comput Biol. 2025 Aug 28;21(8):e1013236. doi: 10.1371/journal.pcbi.1013236 (PMC12393775; doi:10.1371/journal.pcbi.1013236)

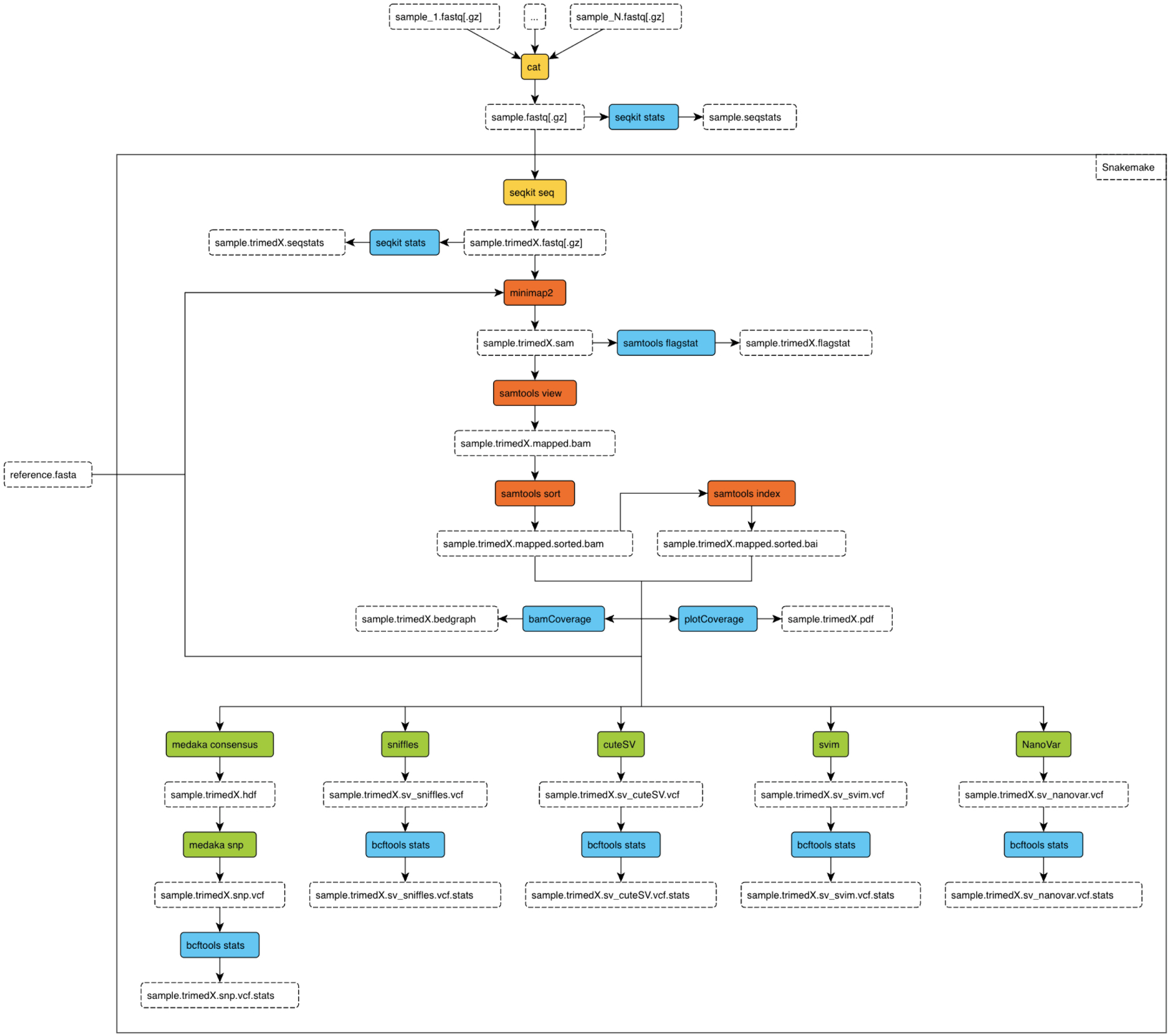

Supplement: S1 Fig — The pipeline has been designed by the supervisors of the project and collaboratively developed with students. The BILL team then continues to maintain and update the pipeline (8). (TIF) [file pcbi.1013236.s001.tif]
